# Diagnostic task shifting for NTDs: outcome of a preliminary quasi-experimental study for microfilaria detection using a novel diagnostic device in Nigeria

**DOI:** 10.4314/ahs.v24i4.7

**Published:** 2024-12

**Authors:** Adeola Onasanya, Temitope Agbana, Opeyemi Oladunni, Jo Van Engelen, Oladimeji Oladepo, Jan Carel Diehl

**Affiliations:** 1 Delft University of Technology, Industrial Design Engineering; 2 AiDx Medical BV; 3 Adeleke University; 4 TU Delft IDE; 5 University of Ibadan College of Medicine, Health Promotion and Education

**Keywords:** NTDs, quasi-experimental study, novel diagnostic device, Nigeria

## Abstract

**Background:**

Lymphatic filariasis (LF) is a Neglected Tropical Disease (NTD) with high morbidity. Tools for detecting LF are either not readily available or used by Community Health Extension Workers (CHEWs) at Primary Health Centers. A newly developed diagnostic device, the AiDx Assist, is targeted for use by CHEWs.

**Objective:**

The study aims to determine the efficiency (speed) and effectiveness (diagnostic capacity) of CHEWs compared to laboratory scientists for detecting LF with the new device, using the World Health Organization's Target Product Profile (TPP) for LF diagnostics as a guide.

**Methods:**

This study utilized a Quasi-experimental design. 7 students undergoing the CHEW program (intervention group) were randomly selected while 2 laboratory scientists (control group) were purposively recruited and were trained to use the device. Thereafter, both groups were tested based on 64 sample slides provided.

**Results:**

The intervention group's efficiency (speed) was similar to the control group. Computed Effectiveness (diagnostic capacity) parameters for the intervention group demonstrated a sensitivity of 85.7% and a specificity of 82.5%.

**Conclusion:**

Given this preliminary result, task shifting to CHEWs for the diagnosis of LF is highly likely to be successful, thereby reducing the prevalence of LF in low-resource settings.

## Introduction

Lymphatic filariasis (LF), one of the Neglected Tropical Diseases (NTDs), is a parasitic infection caused by microscopic worms spread by bites from a range of mosquitoes. It is a cause of significant morbidity including lymphedema, elephantiasis, and hydrocele and is a leading cause of permanent disability and socio-economic loss worldwide^1^,^2^. In 2018, about 51 million people were infected globally^3^ while 341 million Africans are at risk and require intervention^4^. LF is prevalent in Nigeria and the country ranks third globally^5^. The prevalence varies between and within geopolitical zones and numerous studies 6–8 identified a prevalence ranging from 4% in the North-central to 33% in the North-West^9^.

Diagnostic tests for LF include microscopy, polymerase chain reaction (PCR) and antigen tests^2^. Microscopy using a thick film is the usual mode of diagnosis in Nigeria since PCR is expensive, and antigen tests are not always readily available at Primary Health Centers (PHCs)^9^. In addition, the use of microscopy can be challenging in low-resource settings due to the high cost of procuring microscopes for every PHC, and the unavailability of trained personnel to use the microscope^10^.

Nigeria's healthcare structure allows non-physician, non-nurse cadres, such as Community Health Extension Workers (CHEWs) to work in PHCs due to health worker shortages. CHEWs have been a stop-gap to the health worker challenge using a task-shifting approach to healthcare delivery^11^. CHEWs undergo three-year training at Colleges of Health Technology and are the link between communities and the health system. CHEWs are predominant in PHCs serving communities with acute physician and nursing shortages^11^. They are also the first point of call in health posts and small PHCs serving hard-to-reach communities. CHEWs have been trained to make simple diagnoses, undertake simple bedside laboratory tests such as rapid diagnostic tests for malaria, offer treatment for diseases such as malaria, and provide some family planning services^12^. Since LF is predominant in hard-to-reach areas, adequate diagnosis and treatment may not be available due to a lack of skilled personnel for clinical and laboratory diagnosis, thus contributing to the high LF prevalence in Nigeria.

There is a need for task shifting of more complex laboratory processes to CHEWs in other to improve treatment rates, reduce disease burden and reduce the prevalence of LF and other NTDs in Nigeria. There are currently other diagnostic methods being designed for diagnosing NTDs in general and LF specifically, and the World Health Organization (WHO) has developed a Target Product Profile (TPP), which is a guide for diagnostics developers for LF^13^. The TPP guide was used as a guideline to test 3 parameters: the context of use (tool user), performance metrics (specificity and sensitivity) and tool design (throughput and training requirements). The WHO guideline specifies that the diagnostic tool for LF should be easily usable at the primary level of care wherein CHEWs work in Nigeria, require minimal training of less than 1hr, specificity of >78%, sensitivity of >82%, require ≤10 user step, and easily interpreted by untrained eyes. As such, we compared 2 end-users' efficiency and effectiveness using a new diagnostic device, the AiDx assist machine for these 3 components of the TPP.

**Figure 1:**
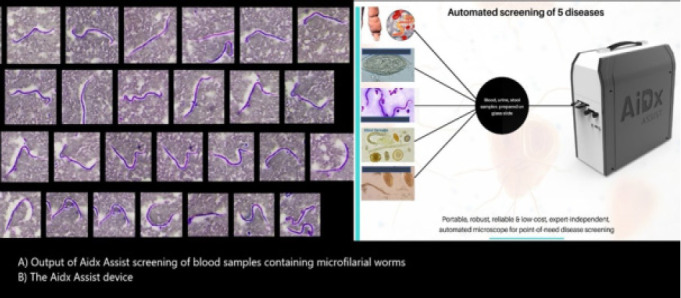
The AiDx Assist Output

The AiDx Assist is a duo-diagnostic system that is developed based on a novel methodology that combines technical optics and specialized data-driven algorithms to realize an integrated, portable, and reliable digital optical diagnostic system for the rapid screening of NTD.^14^ The traditional microscopy-based diagnostic methods have been reported to have sufficiently high specificity necessary for successful elimination programs. However, the cost, need for trained personnel and access to standard microscopes, however, limits the deployment and application of microscopy-based methods for monitoring and surveillance programs. The AiDx has been developed as a fully automated point-of-care device for the specific detection of NTDs such as LF, Schistosomiasis, and Helminthiasis in urine, blood and stool samples accordingly as seen in figure 1^14^.

The expert-independent device is currently optimized to be faster, cheaper, and perform with an accuracy comparable to expert human microscopists. The system output provides additional information on the estimated infection load making it suitable to measure the efficacy of the administered drugs on an infected patient. The generated diagnostic data can be analyzed offline and uploaded onto remote servers. This will accelerate prompt large-scale diagnoses in community mapping for instituting intervention and monitoring. Developed working prototypes have recently undergone low-scale assessments and testing in Nigeria^14^. Furthermore, the diagnostic tool has been optimized for minimal computational effort. As a result, the device, therefore, requires minimal power consumption and it also has an in-built power supply which makes it suitable for use in rural communities with no access to electricity. The robust and portable prototype is usable on the field with limited infrastructure. The usability of the AiDx device in communities where electricity is a challenge makes it a diagnostic tool of choice for the NTD Elimination program.

Prior test conducted in Nigeria with the device has demonstrated 94% sensitivity and a specificity of 99% in the detection of Schistosoma eggs in urine samples compared with microscopy^14^ when used by laboratory scientists. These performance metrics were measured based on a completely automated scan of the prepared sample slides in the x and y direction and the integration of a data-driven autofocus algorithm with an average time for scanning of 8 minutes.

The purpose of the current study was to determine whether the same or similar, results would occur if students undergoing the CHEW program used the AiDx Assist device to make a diagnosis of LF compared to laboratory scientists. The purpose of the current study was to investigate the efficiency (speed) and effectiveness (diagnostic capacity) of students undergoing the CHEW program compared to laboratory scientists to make a diagnosis of LF when using the AiDx Assist device. As such, the study aims to:

Assess if a minimum of 1hr training is sufficient to train students undergoing the CHEW program to use a new diagnostic device
Assess if students undergoing the CHEW program can use a diagnostic device for diagnosing LF after training
Compare the efficiency (speed) of using the devices by students undergoing the CHEW program compared to those of medical laboratory scientists when using the AiDx Assist device

Compare the effectiveness (diagnostic capacity) of students undergoing the CHEW program compared to those of medical laboratory scientists when using the AiDx Assist device.

## Methods

### Study design

This study utilized a Quasi-experimental design that used a control group but no pre-test because we are comparing the efficiency and effectiveness of students undergoing the CHEW program in using a newly created diagnostic device to which they have not been exposed a priori (Static Group Comparison)^15^. A quantitative-structured observational approach was used.

### Study participants

7 students who were undergoing the Community Health Extension Worker (CHEW) training (year 2) were randomly selected from a class of 30 students from the School of Hygiene, Ibadan, Oyo State, Nigeria. Year 2 were selected for the study because they had received training on NTDs and had some laboratory training sessions. Year 3 students (final year) were not available at the time of the study. Trained laboratory scientists/parasitologists with more than 15 years of experience were purposively selected. The students undergoing the CHEW program are the intervention group while the laboratory scientists are the control group.

### Intervention activities

All Participants (students undergoing the CHEW program and laboratory scientists) were taken through a 1-hour training. During the training, knowledge of microfilaria and its detection was elicited and the participants were trained on the use of the AiDx Assist device. The training agenda includes device components, software interface, starting the diagnostic scan, reading the AiDx report visual output and writing out the result. Thereafter, they took a one-hour break After the break, all participants were presented with 64 prepared slides of suspected filariasis to scan using the AiDx device and make a diagnosis.

### Procedure

**Figure 2:**
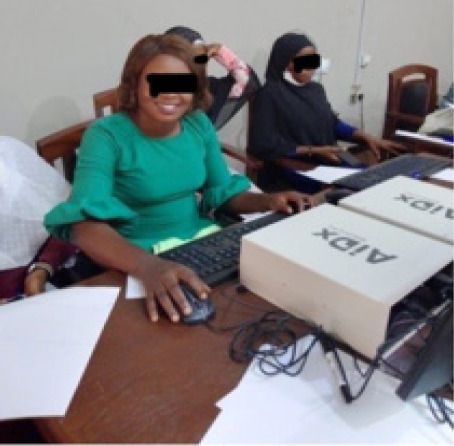
CHEW students using the AiDx

The procedure for the use of the AiDx machine was broken into 3 steps. The CHEW students) were to 1) independently insert the slides into the machine, 2) start an automatic scan, and 3) read, and report AiDx's visual output for the presence/ absence of filarial worms from the AiDx Assist. Thereafter, the fully trained medical laboratory scientists with >15 years of experience followed the 3 steps highlighted above. Any discordant result was validated by comparing them to standard microscopy results. Figure 2 shows a CHEW student using the AiDx device.

### Data

Observational data on AiDx Assist's user efficiency (speed) was collected using a quantitative observational tool. The observation tool involves using a timer to note the time taken to carry out each step of the process of use of the AiDx Assist device by an observer. After the use of the device, the presence /absence of parasites was noted. The effectiveness (diagnostic capacity) was derived by comparing the results written down by the CHEW to those derived by the laboratory scientists. Using a 4*4 table, we calculated the sensitivity, specificity, the negative predictive value. All data were entered into Microsoft Excel software version 2201.

### Consent

Written informed consent was obtained from all participants before enrolment. This study was approved by the UCH/UI Joint Ethical Review Committee, College of Medicine, University of Ibadan. (Reference: UI/EC/21/0641).

## Results

### Sample socio-demography

Seven (7) female students undergoing the CHEW program, age range 20-27 years were recruited. They all use smartphones and have been exposed to the use of microscopes during their training. Two (2) male medical laboratory scientists/ parasitologists with 15 years of experience were recruited who use smartphones and actively use microscopes in their line of work.

### Assessment of training and use

All participants were able to use the AiDx device after a 1-hour training followed by a 1-hour break. CHEW students spent an average of 10.6 minutes using the device compared with laboratory scientists who used an average of 9.8 minutes.

### User Efficiency: time spent per task

With the use of the AiDx machine, the control group (laboratory scientists) were faster than the intervention group (students undergoing the CHEW program) by an average of one minute in total. As regards reading and recording the output of the AiDx machine, the control group was 50% faster than the intervention group. However, the difference was an average of 1 minute. This means that the intervention group's efficiency (speed) of use of the AiDx Assist machine is similar to the user efficiency of the control group as shown in Table 1 and Table 2.

**Table 1:** AiDx Assist user efficiency (speed) of respondents

	Placing a sample(secs)	Starting an automated scan (secs)	Time for scanning (secs)	Reading Output (secs)	Total result (mins)
CHEW program students (Intervention group)	3.1	14.5	480	133.9	10.5
	3.2	16.6	480	136.8	10.6
	3.0	18.00	480	139.6	10.7
	3.1	17.7	480	148.4	10.8
	3.0	20.4	480	130.9	10.6
	3.1	17.63	480	134.1	10.6
	3.2	19.8	480	137.5	10.7
Mean	3.1	17.8	480	137.3	10.6

Laboratory scientists (control group)	2.7	13.8	480	82.4	9.7
	3.2	14.3	480	99.4	9.9
Mean	2.9	14.0	480	90.9	9.80

**Table 2:** Level of significance of user efficiency

	Mean (minutes)	Standard deviation	T statistics	95%CI	Significance level
CHEW students (Intervention group)	10.6	0.2			P < 0.0001*
			17.75	0.711 to 0.889	
Laboratory scientist (control group)	9.8	0.3			

* Significant

### Effectiveness (Diagnostic capacity)

Sensitivity is the ability of a diagnostic test to accurately identify patients with a disease while specificity is the ability of a diagnostic test to correctly identify patients without the disease. When compared with the control group, the intervention group had a sensitivity of about 86% and a specificity of 83% for samples assessed using the AiDx Assist machine. The sensitivity and specificity tests were used to determine the diagnostic accuracy of the intervention group. Students undergoing the CHEW program had an accuracy of 82.8% (71.32-91.10%) compared with laboratory scientists as shown in Table 3.

**Table 3:** Effectiveness (diagnostic capacity) of CHEW program students compared to laboratory scientists

Statistic	Value	95%CI
Sensitivity	85.71%	42.13 - 99.64%
Specificity	82.46%	70.09 - 91.25%
Positive predictive value	37.50%	24.05 - 53.20%
Negative predictive value	97.92%	88.41 - 99.66%
Accuracy	82.81%	71.32 - 91.10%

## Discussion

This study highlights the result of a quasi-experimental study involving the use of an Artificial intelligence-enabled device for diagnosing LF at the primary care level. The AiDx Assist device offers a cost-effective solution for alleviating human resource limitations in primary healthcare settings, as it does not require a significant level of laboratory expertise for use. In addition, diagnostic data can be analyzed remotely, expediting community mapping and intervention.

The results derived from the study indicate that students undergoing the CHEW program were proficient in their capacity to use the AiDx assist device compared to laboratory scientists. The difference in timing for reading results was an average of 1 minute for reading the scanned results, and less than a minute for starting and placing samples and starting at automatic scan. This shows that with adequate training and more exposure to the AiDx Assist machine, CHEWs may replace laboratory scientists in making a diagnosis of lymphatic filariasis at the community level, especially in hard-to-reach areas. It is known that the time taken to make a diagnosis is a challenge with lymphatic filariasis and other NTDs10 as personnel at the health centres and health posts do not have the requisite training for making a laboratory-based diagnosis. Other studies have also corroborated this data concerning the user efficiency (speed) of non-laboratory personnel for laboratory-based work. For instance, task shifting to nurses for HIV testing using a device^16^ and task shifting to non-laboratory personnel using devices to test for clinical chemistry parameters^17^.

The diagnostic capacity (effectiveness) deals with the ability to make an accurate diagnosis using the AiDx device. As regards the diagnostic capacity of the students undergoing the CHEW program, they have a sensitivity of 85.71%, a specificity of 82.46% and an accuracy of 82.8% compared with Laboratory scientists. This indicates that students undergoing the CHEW program can make a laboratory-based diagnosis of lymphatic filariasis and other NTDs at the community level even with minimal training. To the best of our knowledge, this is the first study of its kind to compare laboratory scientists to students undergoing the CHEW program as regards diagnostic performance with the AiDx machine. However, some studies have also compared the diagnostic capacity of laboratory personnel with non-laboratory personnel^18^,^19^. Data from some studies indicate that the competency and capacity for the use of equipment-based near-patient testing are independent of user laboratory qualifications^16^,^17^. Some studies on task shifting have shown that CHEWs can be trained for clinical duties such as treatment of diabetes, hypertension and tobacco cessation^20^–^22^ and HIV with positive results^19^,^23^,^24^. The use of CHEWs is also demonstrated to be cost-effective for the treatment of hypertension in low-resource settings^25^. Other studies have also demonstrated the capability to task shift from laboratory scientists to nurses^16^.

Previous tests conducted in Nigeria using the AiDX assist device have demonstrated impressive results. When used by laboratory scientists, the device showed a sensitivity of 94% and a specificity of 99% in detecting Schistosoma eggs in urine samples compared to traditional microscopy methods^14^. In comparison, students undergoing the CHEW program achieved a sensitivity of 86% and a specificity of 83%. While there is some variation in the effectiveness of the machine's use between the two groups, the overall performance remains quite satisfactory. In addition, based on the WHO requirement of specificity and sensitivity of >78%, and >82% respectively for LF diagnostic devices, it is clear that students undergoing the CHEW program meet the minimum criteria expected for device use effectiveness. This suggests that CHEWs can effectively provide diagnostic laboratory services using artificial intelligence-enhanced tools, thereby contributing to improved healthcare outcomes.

Our results show that CHEW-led diagnostic testing for microfilaria and by extension, other NTDs will ensure decentralization of LF testing, timely return of test results and timely treatment without compromising the quality of testing. This also falls in line with the World Health Organization's (WHO) expectations for Target Product Profiles (TPP) for new diagnostics. It is expected that new diagnostics like the AiDx assist device should be usable by the lowest healthcare cadre, with minimal training that can be given within a day^13^. Given this preliminary result, task shifting for the diagnosis of LF is highly likely. However, achieving this aim will require strong, and consistent training, guidance, and support of CHEWs^26^, and the use of artificial intelligence-supported diagnostic devices is a strong base for task shifting for NTDs.

Some limitations are noted in this study. First, this study only compared the diagnostic capacity of CHEW program students to laboratory scientists using already prepared slide specimens. It did not compare the technical capacity to collect and process samples for analysis. We did not test if sample processing skills may likely limit the capacity of CHEW to use the AiDx machine in low-resource settings. However, Vojnov et al. (2019) have demonstrated that specimen collection was easy to perform and acceptable for non-laboratory staff^19^. Other studies have also demonstrated that non-laboratory personnel can be trained to process samples for analysis^16^,^24^. Second, the recorded speed of reading the diagnostic output of the AiDx machine by both laboratory scientists and CHEW undergoing training may not be as rapid as in non-observed settings compared with this study. The real speed of undertaking the task may likely be slower than observed in this study. Third, the sample size of the prepared slides was small and the diagnostic capacity of CHEW may be different to what was found in this study. Finally, this is a preliminary study and larger studies are currently being planned to validate the result of this study.

## Conclusion

This study has demonstrated that students undergoing the CHEW program were comparable to lab scientists in efficiency (speed) and effectiveness (diagnostic capacity) in detecting blood microfilaria using the AiDx Assist device with good result outcomes after training for one hour. This shows that the technical and experiential requirements for new diagnostic device operations and result interpretation are minimal. Therefore, it is possible for diagnostic task shifting of laboratory process for diagnosing LF to CHEWs thereby supporting the achievement of the WHO 2030 goals for NTDs.
